# Impact of goal-directed fluid therapy on serum cytokines (IL-2, IFN-g, TNF-a, IL-6, IL-8, and IL-10) in elderly patients undergoing thoracotomy

**DOI:** 10.5937/jomb0-54237

**Published:** 2025-11-05

**Authors:** Jia Chen, Lifeng Meng

**Affiliations:** 1 Traditional Chinese Medical Hospital of Zhuji, Department of Anesthesiology, Zhuji, 311800, China; 2 Zhuji People's Hospital of Zhejiang Province, Department of Anesthesiology, Zhuji, 311800, China

**Keywords:** thoracotomy, elderly patients, goal-directed fluid therapy, cytokines, hemodynamic stability, torakotomija, stariji pacijenti, ciljana terapija tečnošću, citokini, hemodinamička stabilnost

## Abstract

**Background:**

This research aimed to investigate the effect of different fluid therapy approaches (conventional and goal-directed fluid management) on TH1 and TH 2 cytokines (IL-2, IFN-g, TN F-a, IL-6, and IL-8, IL-10) during the perioperative period of thoracotomy in elderly patients; as previous studies have only focused on surgical outcomes of goal-directed fluid management.

**Methods:**

Sixty elderly patients who underwent thoracotomy from January 2022 to April 2024 were divided into the control group (CG) and the observation group (OG), with 30 cases in each group. The CG received routine fluid management, while the OG received goal-directed fluid management. The postoperative recovery status, fluid intake and output, hemodynamic indexes, and TH1 and TH 2 cytokine levels were compared between both groups.

**Results:**

The postoperative extubation time, drainage time, ICU stay and hospitalisation time in the OG were reduced than those in the CG (P&lt;0.05). The urine volume, crystalloid volume, colloid volume and total fluid volume in the OG were reduced than in the CG (P&lt;0.05). At 1 h after surgery (T2), the HR and MAP levels of the two groups were reduced than those before surgery (T1) (P&lt;0.05); at the end of surgery (T3), the HR and MAP of the CG were raised than those at T1 (P&lt;0.05); at T2, the HR and MAP levels in the OG were raised than those in the CG (P&lt;0.05); at T3, the HR and MAP levels in the OG were reduced than those in the CG (P&lt;0.05). 1 day after operation, the levels of IL-2, IFN-g, TN F-a, IL-6, and IL-8 were raised than those before surgery, (P&lt; 0.05), and the levels of IL-10 was reduced than those before treatment (P&lt;0.05).

**Conclusions:**

Perioperative goal-directed fluid therapy for elderly patients with thoracotomy is beneficial to maintaining perioperative hemodynamic stability, improving serum levels of TH1 and TH 2 cytokines, reducing the body's inflammatory response, and facilitating early postoperative recovery. This was a novel finding that needs further investigation.

## Abstract (sr)

**Uvod:**

Ova studija je imala za cilj da istraži uticaj različitih pristupa terapiji tečnošću (konvencionalna i ciljana terapija) na TH1 i TH 2 citokine (IL-2, IFN-g, T N F-a, IL-6, IL-8 i IL-10) tokom perioperativnog perioda torakotomije kod starijih pacijenata, budući da su prethodne studije bile fokusirane samo na hirurške ishode ciljane terapije tečnošću.

**Metode:**

Šezdeset starijih pacijenata koji su podvrgnuti torakotomiji od januara 2022. do aprila 2024. godine je podeljeno je u kontrolnu grupu (KG) i grupu za posmatranje (GP), sa po 30 slučajeva u svakoj grupi. KG je primala rutinsko upravljanje tečnošću, dok je GP primala ciljanu terapiju tečnošću. Upoređeni su postoperativni status oporavka, unos i izlučivanje tečnosti, hemodinamički pokazatelji i nivoi TH1 i TH 2 citokina između obe grupe.

**Rezultati:**

Postoperativno vreme ekstubacije, vreme drenaže, boravak na intenzivnoj nezi i vreme hospitalizacije u grupi za posmatranje (GP) je bilo kraće nego u kontrolnoj grupi (KG) (P &lt; 0 ,0 5 ). Volumen urina, volumen kristaloidnih rastvora, volumen koloidnih rastvora i ukupni volumen tečnosti u GP bio je manji nego u KG (P &lt; 0 ,0 5 ). Jedan sat nakon operacije (T2), nivoi srčane frekvencije (HR) i srednjeg arterijskog pritiska (MAP) u obe grupe bili su niži nego pre operacije (T1) (P&lt; 0 ,0 5 ). Na kraju operacije (T3), HR i MAP u KG su bili povišeni u odnosu na T1 (P &lt; 0 ,0 5 ); na T2, HR i MAP u GP su bili viši nego u KG (P &lt; 0 ,0 5 ); na T3, HR i MAP u GP su bili niži nego u KG (P&lt;0.05).

**Zaključak:**

Perioperativna ciljana terapija tečnošću kod starijih pacijenata koji su podvrgnuti torakotomiji je korisna za održavanje hemodinamske stabilnosti tokom perioperativnog perioda, poboljšanje nivoa serumskih TH1 i TH 2 citokina, smanjenje inflamatorne reakcije u telu i olakšavanje ranog postoperativnog oporavka. Ovo je bilo novo otkriće koje zahteva dalja istraživanja.

## Introduction

Thoracotomy refers to a surgical procedure that involves opening the chest or the midline sternum for chest surgery. Depending on the location and approach of the surgery, it can be categorised into median, posterolateral, anterolateral, and minimally invasive thoracotomy, among others [1]. As a significant clinical procedure, thoracotomy trauma often leads to an excessive production of inflammatory cytokines within the body. Perioperative fluid loss and electrolyte imbalance can adversely affect the body, with intraoperative fluid deficiency being particularly detrimental to organ oxygen supply function. This impact is more pronounced in elderly patients.

Elderly patients undergoing thoracotomy are prone to changes in the internal environment and hemodynamic instability due to factors such as the degeneration of various organ functions, multiple underlying diseases, poor vascular elasticity, preoperative bowel preparation, extensive chest exposure during surgery, perioperative fluid loss, and electrolyte imbalance [2]. Additionally, in thoracotomy, single-lung ventilation is often required, increasing the likelihood of postoperative pulmonary oedema in patients. Effective fluid therapy and management during the perioperative period in elderly thoracotomy patients are crucial for stabilising vital signs, ensuring surgical success, and promoting optimal recovery in terms of prognosis [3]. However, excessive fluid supplementation during surgery can lead to local infections, tissue oedema, and anastomotic leakage, impacting the patient's overall recovery [4]. Therefore, determining how to manage perioperative fluids rationally and effectively to stabilise the vital signs of elderly patients, ensure surgical success, and maximise postoperative recovery becomes a significant focus for anesthesiologists [5].

The primary goal of perioperative fluid management is to improve inadequate tissue perfusion and potential circulatory dysfunction while minimising tissue oedema and cardiac dysfunction caused by excessive fluid replacement [6]. Traditional fluid therapy faces challenges in accurately estimating a patient's volume status, often leading to fluid overloading and subsequent bodily volume overload. Moreover, an excessive circulatory blood volume can increase cardiac preload, affecting the hemodynamic stability of the body and potentially leading to fluid rehydration and inadequate tissue perfusion [7]. Targeted fluid therapy adopts a personalised approach to fluid management by monitoring intraoperative hemodynamic parameters such as cardiac index, stroke volume, vascular tone, cardiac preload, and cerebral output indicators. It utilises vasoactive drugs and fluid administration based on individualised fluid needs to maintain optimal vascular tone and cardiac preload. This approach aims to improve the perfusion of body tissues and organs, ensure microcirculation, reduce complications arising from excessive or insufficient circulatory blood volume, and maximise patient outcomes [8] [9]. However, currently, there are relatively few clinical research reports on the application of target-guided fluid therapy. As the current literature highlights the limitations and challenges of traditional fluid therapy in elderly patients undergoing thoracotomy, including the risk of hemodynamic instability, excessive inflammatory response, and poor postoperative recovery, we aimed to investigate the prognostic value of serum IL-2, IFN-γ, TNF-α, IL-6, and IL-8, IL-10 in this population. Furthermore, our study explores the benefits of goal-directed fluid management in maintaining perioperative hemodynamic stability, reducing the body's inflammatory response, and facilitating early postoperative recovery. What makes this study novel is its focus on the specific cytokine profile in elderly patients undergoing thoracotomy and the comparison of traditional versus goal-directed fluid management, providing new insights into the optimal fluid therapy strategy for this high-risk population.

## Materials and methods

### General information

A random number table method was used to divide patients who underwent thoracotomy in our hospital between January 2022 and April 2024 into two groups: the control group (CG) and the observation group (OG). There were 35 patients in each group. Patients in the CG received conventional fluid management, while those in the OG underwent target-guided fluid management. Informed consent was obtained from all patients, and the Hospital's Ethics Committee approved the study. Inclusion criteria were described as follows: (1) patients undergoing thoracotomy; (2) patients aged between 65 and 80 years; and (3) patients with American Society of Anesthesiologists (ASA) physical status classification of I to II, capable of tolerating general anaesthesia. Exclusion criteria were given as follows: (1) patients with contraindications to radial artery catheterisation; (2) patients with cognitive impairment; (3) patients with severe heart, liver, or kidney dysfunction; and (4) patients with incomplete clinical medical records. Table 1

**Table 1:** Demographic characteristics of the study groups.

Variable	CG<br>(Control Group)	OG<br>(Observation Group)	P-value
**Total participants**	**35**	**35**	**-**
Number of males	19	20	0.784
Number of females	16	15	
Age range (years)	65-81	66-79	-
Average age (years) ± SD	71.30±8.64	70.85±7.32	0.091

### Methods of anaesthesia

All preoperative examinations (including routine blood test, routine urine test, coagulation function, liver function, renal function, blood glucose test, acid-base water and electrolyte balance, chest X-ray, electrocardiogram, etc.) were completed in both groups. After entering the room, a multifunctional monitor was opened for routine monitoring of blood pressure, electrocardiogram, pulse oxygen saturation, BIS, etc. The radial artery was punctured and catheterised under local anaesthesia, the arterial blood pressure was monitored continuously, and the upper limb vein was opened. Anaesthesia induction and maintenance were performed in the two groups by a unified method, including oral insertion of a double-lumen bronchial catheter and mechanical ventilation with an anaesthesia machine. After the operation, the endotracheal tube was changed and sent to ICU.

### Liquid management scheme

The CG received conventional fluid management, and the OG received target-directed fluid management. Both groups were given lactate Ringer's fluid (crystal fluid) 8 mL/kg/h as a basic fluid infusion during surgery. The control group received fluid management according to central venous pressure (CVP) and mean arterial pressure (MAP): When CVP was 6-12 mmHg, and MAP was 65-110 mmHg, the regimen of lactate Ringer's solution was continued with 8 mL/kg/h. When MAP <65 mmHg and CVP <6 mmHg were observed, 100 mL of crystal solution was given so that MAP was 65-110 mmHg and CVP was 6-12 mmHg. If not, 50 mL of colloid solution was given for re-evaluation. When MAP < 65 mmHg and CVP >12 mmHg, a small dose of dobutamine (3-5 μg/(kg-h)) was administered intravenously; when MAP >65 mmHg and CVP was 6-12 mmHg, a relatively stable infusion volume was maintained for 5 min for re-evaluation. The observation group received fluid management according to stroke volume variability (SVV) and cardiac index (CI): When CI >2.5 L/min/m^2^ and SVV <1%, the regimen of lactate Ringer's solution was continued with 8 mL/kg/h. When SVV >11% was observed, 50 mL/min hydroxyethyl starch was added until SVV <11%. If fluid was added until SVV <9%, fluid was suspended until SVV >9% was maintained for more than 2 min. After 5 min of hydroxyethyl starch supplementation, if MAP <60 mmHg as well as CI was <2.5 L/min/m^2^ and SVV was still >11%, in order to maintain CI at 2.5-4 L/min/m^2^.

### Observation indicators

Postoperative extubation time, drainage time, hospital stay, drainage time, blood loss, urination volume, crystalloid volume, colloid volume and total fluid volume in 2 groups were observed and recorded. A multifunctional monitor (Draeger, Germany) was used to observe and record the heart rate (HR) and mean arterial pressure before (T1), 1 h (T2) and at the end of operation (T3), respectively.

Fasting venous blood from each group was extracted and separated by a serum separator at 3000 r/min for 10 minutes, and the supernatant was reserved for analysis. IL-2, IFN-γ, TNF-α, IL-6, IL-8 and IL-10 were detected by ELISA. All the kits were bought from Shanghai Enzyme-linked Biotechnology Co., LTD, and instructions were strictly followed.

### Statistical methods

SPSS 20.0 was used for statistical analysis, and count data were compared by χ^2^ test; measurement data were expressed by mean ± standard deviation and comparison in the group was performed by independent T-test. P <0.05 was considered statistically significant.

## Results

In the CG, 19 males and 16 females, aged 65-81, averaged (71.30±8.64) years. In the OG, 20 males and 15 females, aged 66-79, averaged (70.85±7.32) years. General data between both groups showed no difference (P>0.05).

Table 2 compares postoperative recovery metrics between observation and control groups. The metrics include postoperative extubation time (hours), drainage duration (days), hospital stay duration (days), and ICU stay duration (days). The observation group showed significantly shorter postoperative extubation (3.75±1.42 hours vs 6.34±1.55 hours), drainage duration (2.46±0.86 days vs 4.36±1.51 days), and ICU stay (1.41±0.86 days vs 3.02±1.01 days) compared to the control group. The hospital stay duration was also shorter in the observation group, though the difference was less pronounced (9.74±2.70 days vs 11.63±3.85 days). The statistical significance of these differences is reflected in the t-values and p-values, with all p-values being highly significant (p<0.05) except for the hospital stay duration, which is marginally substantial (p = 0.0220).

**Table 2:** Postoperative recovery.

Group	Postoperative extubation (h)	Drainage (d)	Hospital stay (d)	ICU stay (d)
Observation	3.75±1.42	2.46±0.86	9.74±2.70	1.41±0.86
Control	6.34±1.55	4.36±1.51	11.63±3.85	3.02±1.01
*t*	-7.289	-6.469	-2.378	-7.180
*p*	0.000	0.000	0.0220	0.000

Table 3 compares the liquid intake and outflow metrics between observation and control groups. The metrics include blood loss (mL), urine volume (mL/kg/h), crystalloid volume (mL), colloid volume (mL), and total fluid volume (mL). The observation group had a slightly lower blood loss (203.42±36.93 mL vs 212.78±34.88 mL), but this difference was not statistically significant (p = 0.280). However, the observation group had significantly lower urine volume (0.95±0.31 mL/kg/h vs 2.61±0.93 mL/kg/h), crystalloid volume (1227.31 ± 158.01 mL vs 1360.97±170.08 mL), colloid volume (353.25±77.39 mL vs 652.15±106.26 mL), and total fluid volume (1624.31±173.21 mL vs 2095.05±211.24 mL) compared to the control group. These differences are highly statistically significant (p<0.05), as indicated by the p-values.

**Table 3:** Liquid intake and outflow.

Group	Blood loss <br>(mL)	Urine volume <br>(mL/kg/h)	Crystalloid volume <br>(mL)	Colloid volume <br>(mL)	Total fluid volume <br>(mL)
Observation	203.42±36.93	0.95±0.31	1227.31±158.01	353.25±77.39	1624.31±173.21
Control	212.78±34.88	2.61±0.93	1360.97±170.08	652.15±106.26	2095.05±211.24
*t*	-1.090	-10.018	-3.406	-13.452	-10.195
*p*	0.280	0.000	0.001	0.000	0.000

At T1, HR and MAP in both groups were no different (P>0.05); at T2, HR and MAP levels in both groups were reduced than those at T1 (P<0.05). The levels of HR and MAP in the T3 group were raised than those in the T1 group (P<0.05), and HR and MAP levels between the OG and T1 were no difference (P>0.05); at T2, HR and MAP levels in OG were raised than those in CG (P<0.05); at T3, HR and MAP levels in OG were reduced than those in CG (P<0.05), see Table 4.

**Table 4:** Comparison of hemodynamic indexes between the two groups.

Group	HR (n/min)			MAP (mmHg)		
	T_1_	T_2_	T_3_	T_1_	T_2_	T_3_
Observation group	76.22±7.30	68.81±8.62*	74.34±10.67	91.26±7.88	85.16±9.51*	88.40±8.56
Control group	75.32±6.43	62.78±9.54*	81.56±9.54*	90.38±8.64	80.71±8.66*	95.32±7.63*
*t*	0.547	2.775	-2.984	0.445	2.047	-3.570
*p*	0.586	0.007	0.004	0.658	0.045	0.001

Table 5 presents the levels of TH1 cytokines (IL-2, IFN-γ, and TNF-α) in the observation and control groups before and one day after surgery. The observation group showed a significant decrease in IL-2 levels (from 42.56±8.23 pg/mL to 23.42±4.37 pg/mL, P=0.000) and TNF-α levels (from 7.41±2.58 pg/mL to 11.38±3.14 pg/mL, P=0.000) post-surgery, while IFN-γ levels increased significantly (from 14.30±3.12 pg/mL to 20.52±6.82 pg/mL, P=0.004). The control group also experienced significant changes, with a decrease in IL-2 (from 43.21±7.25 pg/mL to 30.37±5.42 pg/mL, P=0.000) and an increase in IFN-γ (from 14.53±3.24 pg/mL to 26.13±8.73 pg/mL, P=0.004) and TNF-α (from 7.82±2.10 pg/mL to 15.42±2.85 pg/mL, P=0.000) levels. However, the changes in the observation group were more pronounced, as indicated by the t-values and P-values, suggesting a more significant impact of the intervention or surgical procedure on cytokine levels in this group.

**Table 5:** TH1 cytokines levels.

Group	IL-2 (pg/mL)		IFN-γ (pg/mL)		TNF-α (pg/mL)	
	Before surgery	1 d after <br>operation	Before surgery	1 d after <br>operation	Before surgery	1 d after <br>operation
Observation<br>group	42.56±8.23	23.42±4.37*	14.30±3.12	20.52±6.82*	7.41±2.58	11.38±3.14*
Control group	43.21±7.25	30.37±5.42*	14.53±3.24	26.13±8.73*	7.82±2.10	15.42±2.85*
*t*	-0.351	-5.906	-0.303	-2.996	-0.729	-5.636
*p*	0.727	0.000	0.763	0.004	0.468	0.000

Table 6 presents the levels of TH2 cytokines (IL-6, IL-8, and IL-10) in the observation and control groups before and one day after surgery. The observation group showed a significant increase in IL-6 levels (from 35.42±8.67 pg/mL to 70.44±12.56 pg/mL, P = 0.000) and IL-8 levels (from 52.68±15.62 pg/mL to 75.62±21.02 pg/mL, P=0.000) post-surgery, while IL-10 levels decreased significantly (from 56.30±11.12 pg/mL to 34.52±9.82 pg/mL, P=0.019). The control group also experienced significant increases in IL-6 (from 35.65±8.46 pg/mL to 84.34±15.92 pg/mL, P=0.000) and IL-8 (from 55.25±14.84 pg/mL to 108.62±25.44 pg/mL, P=0.000) levels, as well as a significant decrease in IL-10 (from 56.53±12.24 pg/mL to 40.13±9.73 pg/mL, P=0.019). However, the changes in the control group were more pronounced for IL-6 and IL-8, suggesting a more significant impact of the intervention or surgical procedure on these cytokines in the control group. 

**Table 6:** TH2 cytokines levels.

Group	IL-6 (pg/mL)		IL-8 (pg/mL)		IL-10 (pg/mL)	
	Before surgery	1 d after <br>operation	Before surgery	1 d after <br>operation	Before surgery	1 d after <br>operation
Observation<br>group	35.42±8.67	70.44±12.56*	52.68±15.62	75.62±21.02*	56.30±11.12	34.52±9.82*
Control group	35.65±8.46	84.34±15.92*	55.25±14.84	108.62±25.44*	56.53±12.24	40.13±9.73*
*t*	-0.112	-4.055	-0.706	-5.916	-0.082	-2.401
*p*	0.911	0.000	0.483	0.000	0.935	0.019

## Discussion

This study compared conventional fluid management with target-guided fluid management in elderly thoracotomy patients, revealing that target-guided fluid therapy (TFT) significantly reduced extubation, drainage, ICU, and hospital stay durations, as well as urine output and total fluid volume, suggesting improved postoperative recovery and hemodynamic stability, similar to Gopal et al., [10] study. TFT maintained lower heart rate (HR) and mean arterial pressure (MAP) levels both at the end of surgery and postoperatively, closer to preoperative levels, indicating better hemodynamic control. TFT, guided by indicators such as stroke volume variation (SVV), cardiac index (CI), and cardiac output (CO), effectively monitors and optimises fluid input, enhancing vasoactive drug efficacy and preventing volume overload or insufficiency [10] [11] [12]. Furthermore, TFT reduced postoperative inflammatory cytokine levels (IL-6, IL-8, IL-2, IFN-γ, and TNF-α) and increased anti-inflammatory cytokine (IL-10) levels, suggesting a reduced inflammatory response and improved postoperative recovery, likely due to better hemodynamic stability and effective microcirculation perfusion [10] [13] [14] [15] [16] [17] [18] [19].

Jin et al. [20] conducted a systematic review and meta-analysis to evaluate the impact of GDFT on perioperative outcomes after oncologic surgeries. Jin et al. [20] found that GDFT significantly reduced the length of hospital stay by 1.57 days (P<0.01). Similarly, our study observed a reduction in hospitalisation time in the observation group (P<0.05), highlighting the potential of GDFT to expedite recovery and reduce hospital burden. Jin et al. [20] reported a lower incidence of postoperative complications in the GDFT group (risk ratio, 0.74; P=0.03). Our study also noted fewer complications, as indicated by reduced extubation time, drainage time, and ICU stay in the observation group (P<0.05). Jin et al. [20] did not focus on the inflammatory response, but our study found that GDFT modulated the inflammatory response. But Funk et al. [19] conducted a randomised controlled trial to assess the effects of GDFT on the inflammatory response in patients undergoing open abdominal aortic aneurysm repair. Funk et al. reported fewer postoperative complications in the GDFT group (P=0.02), consistent with our findings of improved recovery and reduced complications in the observation group. Unlike our study, Funk et al. did not find a significant difference in the levels of inflammatory cytokines between the GDFT and control groups. This suggests that the clinical benefits of GDFT may not be directly related to a modulation of the inflammatory response, which is a different conclusion from our study.

Our study aligns with the findings of Wodack et al. [18] and Obradovic et al. [16] but also introduces novel insights. Wodack et al. [18] focused on optimising intravascular fluid status during the early course of severe acute pancreatitis, showing that individualised goal-directed therapy led to less vascular endothelial damage, pancreatic oedema, and reduced inflammatory response.

In contrast, Obradovic et al. [16] compared the effects of goal-directed crystalloid versus colloid administration on perioperative inflammatory markers in patients undergoing moderate to high-risk abdominal surgery. They found no significant differences in cytokine levels between the two groups, contrasting our findings. In our study, goal-directed fluid therapy significantly reduced IL-2, IFN-γ, TNF-α, IL-6, and IL-8 while increasing IL-10 levels, indicating a more balanced TH1/TH2 cytokine profile. This suggests that the specific context of thoracotomy in elderly patients may elicit a different cytokine response than abdominal surgery and that goal-directed therapy may have a more pronounced effect on modulating the immune response in this population.

Gu et al. [21] further expand the comparison by evaluating the effect of goal-directed fluid therapy guided by different goals on endothelial glycocalyx and inflammatory cytokines during high-risk abdominal surgery. They found that GDFT with a target of SVV 9%-14% was more effective in reducing endothelial glycocalyx degradation and endothelial barrier damage than SVV <9%. This aligns with our findings, where goal-directed fluid therapy maintained hemodynamic stability and modulated the immune response, as evidenced by reduced inflammatory cytokine levels and increased IL-10 levels. The alignment of these results suggests that the specific goals used in goal-directed fluid therapy can significantly influence perioperative outcomes and that a more stringent goal (SVV 9%-14%) may benefit elderly thoracotomy patients.

### Study limitations

This study has several limitations. First, the sample size is relatively small, which may limit the generalizability of the findings to a broader population. Second, the study only evaluated cytokine levels and hemodynamic parameters but did not include a comprehensive assessment of long-term outcomes such as mortality and quality of life. Third, the mechanisms by which GDFT influences cytokine levels and inflammatory responses remain unclear and warrant further investigation. Lastly, the study did not control for potential confounding factors such as preoperative health status and comorbidities, which could affect the results. These issues could be improved and addressed in further research.

## Conclusion

In conclusion, target-directed fluid therapy for elderly patients undergoing thoracotomy during the perioperative period is beneficial for maintaining perioperative hemodynamic stability, improving serum TH1 and TH2 cytokines levels, alleviating inflammatory response, and facilitating early postoperative recovery of patients.

## Dodatak

### Funding

The research is supported by a medical science research project in Hebei province and the clinical application of target-directed fluid therapy in senile thoracic surgery (No. 20200317).

### Author contribution

Jia Chen and Lifeng Meng contributed equally to this work as co-first authors.

### Conflict of interest statement

All the authors declare that they have no conflict of interest in this work.

## References

[1] Zengin M, Alagoz A (2021). Comparison of Thoracic Epidural Analgesia and Thoracic Paravertebral Block Applications in the Treatment of Acute Pain After Thoracotomy in Geriatric Patients. Cureus.

[2] Ulger G, Baldemir R (2021). Comparison of Patient-Controlled Analgesia With Tramadol or Morphine After Video-Assisted Thoracoscopic Surgery in Geriatric Patients. Cureus.

[3] Liu X, Zhang P, Liu M X, et al (2021). Preoperative carbohydrate loading and intraoperative goal-directed fluid therapy for elderly patients undergoing open gastrointestinal surgery: a prospective randomized controlled trial. BMC Anesthesiol.

[4] Ali-Osman F, Mangram A, Sucher J, et al (2018). Geriatric (G60) trauma patients with severe rib fractures: Is muscle sparing minimally invasive thoracotomy rib fixation safe and does it improve post-operative pulmonary function?. Am J Surg.

[5] Haas S, Eichhorn V, Hasbach T, et al (2012). Goal-Directed Fluid Therapy Using Stroke Volume Variation Does Not Result in Pulmonary Fluid Overload in Thoracic Surgery Requiring One-Lung Ventilation. Crit Care Res Pract.

[6] Chong M A, Wang Y, Berbenetz N M, et al (2018). Does goal-directed haemodynamic and fluid therapy improve peri-operative outcomes?: A systematic review and meta-analysis. Eur J Anaesthesiol.

[7] Joosten A, Coeckelenbergh S, Alexander B, et al (2020). Hydroxyethyl starch for perioperative goal-directed fluid therapy in 2020: a narrative review. BMC Anesthesiol.

[8] Aaen A A, Voldby A W, Storm N, et al (2021). Goal-directed fluid therapy in emergency abdominal surgery: a randomised multicentre trial. Br J Anaesth.

[9] Wrzosek A, Jakowicka-Wordliczek J, Zajaczkowska R, et al (2019). Perioperative restrictive versus goal-directed fluid therapy for adults undergoing major non-cardiac surgery. Cochrane Database Syst Rev.

[10] Gopal J, Srivastava S, Singh N, et al (2023). Pulse Pressure Variance (PPV)-Guided Fluid Management in Adult Patients Undergoing Supratentorial Tumor Surgeries: A Randomized Controlled Trial. Asian J Neurosurg.

[11] McLain N, Parks S, Collins M J (2021). Perioperative Goal-Directed Fluid Therapy: A Prime Component of Enhanced Recovery After Surgery. AANA J.

[12] Joosten A, van der Linden P, Vincent J L, et al (2021). Goal-directed fluid therapy for oesophagectomy surgery. Br J Anaesth.

[13] Wang X, Wang N, Wang X, et al (2021). Application value of goal-directed fluid therapy with ERAS in patients undergoing radical lung cancer surgery. Am J Transl Res.

[14] Wang X, Duan Y, Gao Z, et al (2021). Effect of Goal-directed Fluid Therapy on the Shedding of the Glycocalyx Layer in Retroperitoneal Tumour Resection. J Coll Physicians Surg Pak.

[15] Xu H, Shu S H, Wang D, et al (2017). Goal-directed fluid restriction using stroke volume variation and cardiac index during one-lung ventilation: a randomized controlled trial. J Thorac Dis.

[16] Zhang N, Liang M, Zhang D D, et al (2018). Effect of goal-directed fluid therapy on early cognitive function in elderly patients with spinal stenosis: A Case-Control Study. Int J Surg.

[17] Funk D J, HayGlass K T, Koulack J, et al (2015). A randomized controlled trial on the effects of goal-directed therapy on the inflammatory response open abdominal aortic aneurysm repair. Crit Care.

[18] Jin Z, Razak A, Huang H, Muthukumar A, Murphy J, Shteynman L, Bergese S D, Gan T J (2022). Intraoperative Goal-Directed Fluid Therapy and Outcomes After Oncologic Surgeries: A Systematic Review and Meta-Analysis. Anesthesia & Analgesia.

[19] Wodack K H, Poppe A M, Lena T, Bachmann K A, Strobel C M, Bonk S, Havel J, Heckel K, Gocht A, Saugel B, Mann O (2014). Individualized Early Goal-Directed Therapy in Systemic Inflammation: Is Full Utilization of Preload Reserve the Optimal Strategy?. Critical Care Medicine.

[20] Obradovic M, Kurz A, Kabon B, Roth G, Kimberger O, Zotti O, Bayoumi A, Reiterer C, Stift A, Fleischmann E (2020). The effect of intraoperative goal-directed crystalloid versus colloid administration on perioperative inflammatory markers - a substudy of a randomized controlled trial. BMC Anesthesiology.

[21] Gu J, Gao Z, Wang X, Ding L, Yao L (2018). Effect of goal-directed fluid therapy guided by different goals on endothelial glycocalyx in patients undergoing high-risk abdominal surgery: a prospective randomised controlled clinical study. Chinese Journal of Anesthesiology.

